# Instructional design framework for the sex and gender-specific health multimedia case-based learning modules

**DOI:** 10.1186/s13293-016-0095-5

**Published:** 2016-10-14

**Authors:** Steven M. Crooks, Jongpil Cheon, Robert Casanova, Marjorie Jenkins

**Affiliations:** 1 Department of Organizational Learning and Performance, College of Education, Idaho State University, Stop 8081, Pocatello, ID 83209-8081 USA; 2 Department of Educational Psychology and Leadership, College of Education, Texas Tech University, Box 41071, Lubbock, TX 79409 USA; 3 Obstetrics and Gynecology, Texas Tech University Health Sciences Center, 3601 4th St, Stop 8326, Lubbock, TX 79430 USA; 4 Laura W. Bush Institute for Women’s Health, Texas Tech University Health Sciences Center, Amarillo, TX 79106 USA

**Keywords:** Sex and gender, Sex and Gender Specific Health, Case-based learning module

## Abstract

The goal of the Sex and Gender Specific Health (SGSH) curriculum at the Texas Tech University Health Sciences Center (TTUHSC) is to advance the understanding of sex/gender differences, increase the awareness of gender-specific health issues, and improve the knowledge of sex and gender evidence-based medicine. The purpose of this paper is to explain the development and theoretical rationale for an important aspect of the curriculum: the SGSH Multimedia Case-Based Learning Modules (MCBLMs). The MCBLMs are designed to be used throughout the TTUHSC curriculum as a stand-alone or a supplementary instructional resource. The MCBLMs provide students with authentic learning opportunities that integrate the learning of SGSH with more traditional clinical knowledge and skills. The MCBLMs are specifically designed to enhance students’ clinical reasoning and decision-making skills by portraying realistic clinical scenarios. In this way, students are able to practice effective SGSH as competent health-care professionals.

## Background

A compelling amount of evidence now suggests that many diseases common to both women and men contain sex or gender differences in etiology, pathology, presentation, and treatment response [1]. Unfortunately, much of the evidence-based information about sex or gender differences is not applied in clinical practice [2]. It is vital to understand and apply these differences in the clinical care process in order to improve health-care outcomes for both women and men; this is the primary purpose of sex and gender-specific health (SGSH) initiatives in clinical medicine [3, 4]. Several initiatives have been implemented to identify and address SGSH differences in health care. However, most of these initiatives have been short in-service training workshops; very few initiatives have involved sustained pre-service training interventions for medical, nursing, or public health professionals [2, 5].

To address the need for SGSH education in medical school curricula, the Laura W. Bush Institute for Women’s Health at the Texas Tech University Health Sciences Center (TTUHSC) accepted the task of integrating SGSH education into the existing TTUHSC curricula. An important component of this educational initiative was the development of the SGSH Multimedia Case-Based Learning Modules (MCBLMs). The overarching goal of the MCBLMs was to provide faculty with curricular threads (i.e., the MCBLMs) for weaving SGSH content into the fabric of their current curricula as stand-alone or supplementary resources. A SGSH curriculum committee (consisting of faculty, students, and instructional design experts) was formed to accomplish this goal.

The committee began their work by identifying factors they considered critical to creating engaging curriculum (i.e., writing clear instructional goals, selecting important subject matter, and identifying optimal instructional delivery media) and instruction (i.e., selecting a theory-based instructional framework for engaging instruction). In the next section, we discuss the MCBLM *curricular framework* (i.e., goals, subject content, and media). In the remaining sections, we discuss problems associated with traditional lecture-based approaches to medical education and how the *instructional framework* for the MCBLMs was designed to address these problems, that is, how contemporary theories of instruction guided our selection of specific MCBLM instructional features.

## The MCBLM curricular framework: goals, content, and media

The SGSH curriculum committee identified the following goals to guide the design of the MCBLMs:

- Increase awareness of gender-specific health issues
- Advance understanding of sex/gender differences
- Improve knowledge of sex/gender evidence-based medicine
- Demonstrate the benefits of an interprofessional approach to health care
- Engage students in real-world medical problem-solving

After identifying the goals of the MCBLMs, the committee deliberated on the SGSH content that they considered most important for medical professionals to understand. The instructional content was selected by identifying specific diseases that contain SGSH differences. An important criterion for selecting instructional content was the prominence of SGSH differences within a specific disease state. The following diseases were selected as the basis for the instructional content of the MCBLM modules:

- Osteoporosis
- Diabetes
- Cardiovascular disease
- Alcohol addiction
- Infectious disease

The SGSH curriculum committee realized the importance of selecting the appropriate delivery media for the SGSH content. The importance of this decision was evident as the committee considered the unique context and manner that the SGSH instruction was to be implemented. For example, the SGSH instruction was to be made available to faculty to use at their discretion as “curricular threads,” able to be woven throughout the existing curricula. This implementation constraint required the SGSH content to be chunked into small modules that could be used to supplement a variety of existing lessons occurring throughout a curricular block.

Another implementation constraint was the need for the SGSH content to be accessible to faculty as stand-alone instructional modules. That is, the modules must not require the presence of an instructor to facilitate the learning experience; the modules must “teach” the SGSH content by providing both the content and the instructional methods needed for optimal student learning about a given content area. A final implementation constraint was the need to provide authentic learning experiences that integrate the SGSH content with traditional clinical knowledge and skills.

After considering the implementation constraints mentioned above, the committee determined that a computer-based delivery medium would satisfy both the “small chunks” and the “stand-alone” constraints. The committee also determined that a computer, with multimedia capabilities, would be an ideal medium for addressing the “authentic learning” constraint. Multimedia instruction has the capability of providing realistic scenarios that mimic real-world situations.

## The MCBLM instructional framework

Traditional instructional approaches in medical schools often fail to adequately prepare students to apply medical knowledge, such as SGSH [6], in clinical settings due to the following reasons:

- A preponderance of lecture-based classroom learning tends to promote the acquisition of a broad range of content knowledge (e.g., the basic sciences) to the exclusion of *situational* and *strategic* knowledge. Content knowledge is difficult to apply in clinical settings without the acquisition of these other important knowledge areas [7].
- Clinical settings are ideal for developing situational and strategic knowledge, but the separation (in space and time) between clinical and classroom instruction makes it difficult for students to connect content knowledge with situational and strategic knowledge [7]. These are typical challenges in higher education due to the differences between classroom problem-solving (well-structured problem-solving) and real-world problem-solving (ill-structured problem-solving).
- The knowledge learned in clinical settings is largely dependent upon the medical cases available at specific times. This is especially problematic with SGSH education because the ideal learning situation is dependent on the unlikely occurrence of male and female patients presenting identical symptoms at the same clinic and the same time [8].

To overcome the challenges mentioned above, the SGSH MCBLMs were designed based on an evidence-based pedagogical framework that has proven to enhance problem-solving and decision-making abilities in dynamic, real-world settings. The SGSH committee realized that to train medical professionals to function effectively in a health-care environment, an instructional program needs to contain more than isolated facts and “how-to’s” or even sophisticated role-plays. The essence of a medical professional’s job cannot be experienced piecemeal by breaking it apart and studying each component in isolation, as is often seen in medical school lectures and ground-rounds presentations [9].

An interactive case-based learning approach should enable students to simultaneously apply medical knowledge and skills without compartmentalizing knowledge areas as separate entities to be learned independently. Some might argue that if immersing medical students in an authentic medical setting is optimal, then why not train them in an actual medical facility with real medical equipment and patients? There are three problems with this approach:

1. The price of mistakes are too high in a real health-care environment. Even routine mistakes or errors in judgment can (and do) cost health-care institutions millions of dollars. Case-based learning provides medical students with opportunities to make mistakes (and learn from them) without compromising patient care.
2. A real health-care environment provides instructors with minimal control over the complexity, content, and pacing of the learning experience. A case-based learning environment provides instructional designers with more control of the learning experience, thereby ensuring that learners experience the right content at the right time during the learning process. This approach avoids the problem of cognitive overload that medical students can experience when placed in an actual medical situation without prior training.
  Control over the learning experience is especially important in SGSH because the nature of the subject matter requires a compare and contrast instructional method that is difficult to implement in a real medical setting; studying SGSH requires students to compare and contrast the onset and progression of disease as expressed in different populations (i.e., male and female). A case-based learning approach enables the instructional designer to juxtapose contrasting cases in a manner that highlights important SGSH differences.
3. The actual health-care environment is almost never conducive to a quality learning experience for new medical students. The noise level and stress associated with the need to make appropriate medical decisions in a real health-care environment often takes away from the quality of the learning experience. This is especially the case with new medical students. A case-based approach allows new students to use all of their cognitive resources in learning the substantive aspects of their profession without having to deal with less substantive or even trivial aspects before they have learned the basics.

## The design rationale

Common sense suggests that effective medical education should consist of instructional practices that are grounded in research-based theories about how people learn [10]. Separating the science of learning (i.e., how people learn) from the design of instruction is analogous to separating the science of medicine from the practice of medicine. The need for evidence-based medical education is especially important in an age when changes in health-care delivery and advances in medicine pressure medical schools to expand the scope and depth of their curricula [11]. And yet, the practice of medical education is often disconnected from the science of learning. For instance, in their meta-analysis of instructional interventions in medical education, Cook et al. [12] found scant evidence that instructional programs were guided by evidence-based principles of learning and instruction. Too much is at stake for medical schools to ignore current research-based theory as a foundation for instructional practice.

The theoretical framework we chose for the design of the MCBLMs is the cognitive-affective theory of learning with media (CATLM) [13, 14]. Two assumptions from the CATLM guided the design of our intervention: (a) deep, meaningful learning occurs when learners invest cognitive effort in purposefully integrating new information with existing knowledge and (b) motivational factors mediate learning by increasing or decreasing cognitive effort. The CATLM enhances traditional multimedia learning theories (e.g., Mayer’s cognitive theory of multimedia learning [15]) by expanding the traditional cognitive perspective to include affective and motivational aspects of learning.

As mentioned previously, the Cook et al. [12] meta-analysis found few educational interventions that were guided by learning theory; further, those interventions that were guided by theory did not intentionally consider motivational theory in their designs. This is a significant oversight considering the significant evidence showing that motivational and cognitive processes are inextricably interwoven in learning [16, 17]. We considered motivation to be a particularly crucial instructional design component in SGSH education because its novelty in medical education makes it susceptible to being passed over for topics considered by many to have higher priority.

Applying the CATLM to the design of the MCBLMs enabled us to add to the design of existing multimedia medical programs by purposefully combining cognitive and motivational design elements into our instructional intervention. In this way, we address both the cognitive and motivational needs of medical students. We now discuss how we addressed both cognitive and motivational learning needs in the MCBLMs.

## Cognitive aspects: types of knowledge essential to SGSH education

A central cognitive tenet of the CATLM is that students must learn to integrate new information with their existing knowledge before they can apply knowledge to real-world problems. More specifically, in order for medical students to transfer diagnostic reasoning to medical situations, they must integrate (a) *content knowledge*, (b) *situational knowledge*, and (c) *strategic knowledge* [18–21] with their existing knowledge. Unfortunately, traditional medical education has tended to overemphasize content knowledge while underemphasizing situational and strategic knowledge. Nevertheless, all three types of knowledge are essential to appropriately address patient-care issues related to SGSH. In the following sections, we describe each knowledge type, explain its role in developing deep, meaningful learning, and present evidence-based instructional approaches that have been found to facilitate each knowledge type. We then describe how specific aspects of the MCBLMs were designed to facilitate the development of each knowledge type.

### Content knowledge

The content knowledge associated with SGSH education includes the facts, concepts, principles, and procedures that undergird this knowledge domain. Choi and Hannafin [22] recommend that designers consider content diversity and anchored instruction as ways of embedding meaningful content into authentic learning environments. Content diversity can be achieved by varying the situations in which students practice what they have learned [23]. This helps students to learn at a level of generality that enables them to transfer knowledge and skills to new situations. Anchored instruction refers to problem-rich environments that encourage exploration and diversity of perspectives. Providing students with macro-contexts is one way to anchor instruction, so that learners can see and explore the interrelationships among content knowledge (e.g., relationships between SGSH concepts and principles). The Jasper Series, for example, provides students with contextually anchored videodisk mathematics and science problem-solving tasks. Students participating in these units can see the interrelationships between mathematical formula and scientific exploration [24].

### Situational knowledge

Situational knowledge involves knowledge about the cultural and social contexts of real-world situations, problems, and activities. This knowledge represents the milieu in which content knowledge (e.g., facts, concepts, principles) is applied in the real world [25]. Medical students must understand the dynamic cultural and social contexts of a medical situation before they can effectively apply medical content knowledge [26]. The implication for medical education is that *what* is learned (e.g., facts, concepts, principles) should not be separated from the *context* in which it is intended to be used. One example of situational knowledge in heath care is understanding how patients’ cultural backgrounds can influence their perceptions about the cause of disease (e.g., punishment from God, the actions of others) and how these perceptions can influence individual differences in coping with disease and bereavement [27]. This knowledge has obvious implications for appropriate interactions between health-care workers, patients, and their families.

Instructional environments such as problem and case-based learning have been found to facilitate students’ acquisition of situational knowledge. These environments are characterized by authentic tasks, the kinds of tasks performed by practitioners in *real* problem-solving situations. The authenticity of a task can be enhanced by embedding the task within cultural and social contexts similar to the real world. This enables students to reason like practitioners and to use contextual information to help them solve problems. The situational knowledge produced by these methods contrasts sharply with the abstract, decontextualized knowledge often produced in more formal educational settings [22, 28].

The development of situational knowledge also has important implications for assessment of student learning. Choi and Hannafin [22] recommend performance assessment as an effective way of measuring situated knowledge outcomes. Performance assessments involve asking students to produce things or to perform tasks that have some direct connection to the real world. Many researchers feel that standardized tests, criterion-referenced tests, and teacher-constructed tests do not adequately measure many important learning outcomes such as the degree of student understanding or the quality of their thinking process [29, 30]. Authentic assessment activities, such as performance assessment, not only represent valid assessments of situated knowledge, but they also contribute to the development of situational knowledge.

### Strategic knowledge

While situational knowledge involves contextual understanding of real-world situations, strategic knowledge involves understanding how to *use* this knowledge [31]. Situational knowledge involves knowing *what*; strategic knowledge involves knowing *how*. This includes knowing how to use knowledge in new contexts, knowing how to reflect on plans and actions performed, and developing tacit knowledge. This tacit knowledge is characteristic of an expert’s ability to use domain knowledge (e.g., facts, concepts, and procedures) to solve problems within their area of expertise [31].

Choi and Hannafin [22] recommend providing students with scaffolding to support their strategic problem-solving and decision-making attempts. Scaffolding can take the form of any method, resource, or tool that supports novice learners in developing a deeper understanding of knowledge that is initially beyond their capacity to understand and apply [32]. One example of scaffolding students’ understanding of domain knowledge is the *contrasting cases* method [33, 34]. This method is designed to guide and focus student attention on the salient aspects of an expository text or lecture.

One reason that texts and lectures are often ineffective learning methods is that students have insufficient background knowledge to effectively direct their attention toward salient information that needs to be integrated with existing knowledge. Without sufficient background knowledge, students often memorize surface information rather than relate complex conceptual knowledge with their prior knowledge [35]. This obviously hampers students’ abilities to develop knowledge essential for diagnostic reasoning and problem-solving.

Contrasting cases involve juxtaposing carefully chosen dissimilar scenarios to highlight distinctions between cases that students might otherwise overlook [33]. When contrasting cases have been employed before, rather than after, reading text or listening to a lecture, students can developed a robust understanding of the material [33, 34]. This method is particularly germane to SGSH education because of the compare and contrast nature of this knowledge domain.

### Implementation of Knowledge Types in the SGSH modules

The MCBLMs emphasize situational knowledge by immersing learners in virtual patient-care situations. These virtual cases enable students to develop knowledge of concepts as they relate to particular medical situations. These case-based learning environments exhibit the same major elements and constraints typically experienced by medical professionals in their actual work environment. Each video case provides a learning environment that mimics a real medical situation involving a sex and gender-based issue.

The authenticity of the SGSH learning modules is preserved by introducing each medical case with patients interacting with medical care professionals about a medical problem. Patients and medical professionals are represented visually on a computer screen as animated characters who interact verbally via the voice recordings of professional voice actors. Medical students are further engaged in the SGSH cases by an on-screen guide (an aviator playing the role of a senior medical resident) who invites them to assume the role of a medical professional tasked with developing an appropriate medical care plan for each patient case.

After watching an SGSH case, the on-screen guide instructs the medical student to contact medical experts (again played by on-screen avatars) who can provide them with information needed to develop appropriate patient-care plans. The experts portray real medical professionals whose jobs require different medical expertise (e.g., basic scientists, practicing physicians, nurse practitioners). This further preserves the authenticity of the SGSH cases by requiring students to obtain the knowledge needed to solve the patient cases by contacting medical professionals known to possess the requisite knowledge in the real world.

The SGSH models incorporate authentic assessment by requiring students to perform medical diagnosis and recommend treatment plans in realistic patient-care situations. This process affords educators the ability to collect authentic assessment data related to the effectiveness of students’ performance based upon actual module goals. This form of assessment has high validity because the context of assessment is virtually the same as the context of the actual patient-care setting. Transfer of learning is also higher than in traditional classroom-based medical education because the students learn to focus their efforts on mastering authentic skills that are easily transferred to patient-care situations.

In addition to authentic assessment, the SGSH modules incorporate more traditional assessment items with feedback to help students master prerequisite knowledge in areas such as the basic medical sciences. This combination of assessment techniques provides each student with a rich database of information about their performance as a medical professional.

The SGSH modules contain robust usable content knowledge. This knowledge is developed by forming and utilizing subject-matter content committees in the design of each module. These committees consist of a diverse group of medical experts who can provide unique perspectives on each patient case.
